# Circulating MicroRNAs as Clinical Biomarkers in the Predictions of Pregnancy Complications

**DOI:** 10.1155/2015/294954

**Published:** 2015-01-29

**Authors:** Marthe Tsochandaridis, Laurent Nasca, Caroline Toga, Annie Levy-Mozziconacci

**Affiliations:** ^1^Assistance Publique-Hôpitaux de Marseille, Laboratoire de Biochimie et Biologie Moléculaire, Unité de Biologie Materno-Fœtale, Hôpital Nord, Chemin des Bourrely, 13015 Marseille, France; ^2^Assistance Publique-Hôpitaux de Marseille, Centre Pluridisciplinaire de Diagnostic Prénatal, Hôpital Nord, Chemin des Bourrely, 13015 Marseille, France

## Abstract

Predicting pregnancy complications is a major topic for clinicians and biologists for maternal and fetal monitoring. Noninvasive biomarkers in maternal blood such as circulating microRNAs (miRNAs) are promising molecules to predict pregnancy disorders. miRNAs are noncoding short RNAs that regulate mRNA expression by repressing the translation or cleaving the transcript. miRNAs are released to the extracellular systemic circulation via exosomes. The discovery of plasma- or serum-derived miRNAs and of free-circulating exosomes that contain miRNAs provides useful information about the physiological or pathophysiological roles of the miRNAs. Specific placental miRNAs are present in maternal plasma in different ways depending on whether the pregnancy is normal or pathological or if there is no pregnancy. This paper focuses on placental miRNAs and extracellular miRNAs to the placenta whose misregulation could lead to pregnancy complications.

## 1. Introduction

The discovery of fetal DNA in maternal blood has opened new perspectives on noninvasive prenatal diagnosis development [1]. Since then, other fetal specific nucleic acids related to placenta circulating throughout the plasma and the serum of pregnant women have been found: mRNA [2] and miRNA [3]. Specific placental miRNAs circulating through the plasma reflect the physiological state of pregnancy and are undetectable after delivery [4]. This suggests that their concentration and their profiles in the plasma make them possible candidates as biomarkers for the detection of pregnancy complications linked to placental pathologies. The identification of specific placental miRNAs in maternal blood as noninvasive biomarkers in pregnancy management is developing rapidly. Early noninvasive diagnosis techniques could facilitate medical monitoring for both the mother and the fetus. In this paper, we review the misregulated expression of circulating placental miRNAs related to specific pregnancy pathologies. These molecules could be promising biomarkers for noninvasive diagnosis or the prediction of preeclampsia, intrauterine growth restriction, and early pregnancy loss.

## 2. MicroRNAs

**Figure 1:**
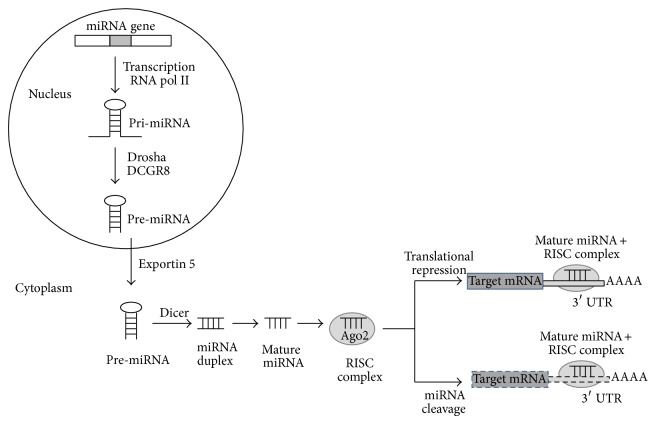
miRNA biogenesis. miRNA gene is transcribed by RNA pol II to form a hairpin loop primary transcript, pri-miRNA, which is processed by Drosha/DCGR8 to form pre-miRNA. Pre-miRNA is exported to the cytoplasm by exportin 5 where Dicer cleaves off the hairpin loop to form a duplex that contains the mature miRNA. The mature miRNA is then incorporated into the RNA-induced silencing complex RISC to target the 3′ untranslated region of the target mRNA to silence expression by repression or cleavage.

In the early 2000s the term microRNA (miRNA) was first introduced. MicroRNAs are small noncoding RNAs about 20–24 nucleotides long. These molecules have been shown to play critical regulatory roles in a wide range of biological and pathological processes [5]. miRNAs regulate cellular gene expression at the posttranscriptional level by suppressing the translation of protein coding genes. This process is made possible by the perfect pairing of miRNAs and mRNAs or their imperfect pairing leading to cleft mRNAs of protein coding genes. In both cases the pairing is performed at 3′UTR (untranslated region) or 5′UTR of mRNA [6]. The miRNA biogenesis is a multistep complex process (Figure 1): miRNA genes are transcribed as primary miRNA (pri-miRNA) in the nucleus by the enzyme RNA pol II. RNA pol II is bound to the promoter region of a specific DNA sequence and forms the pri-mRNA which presents a hair pin structure. This pri-miRNA transcript is processed by the Drosha endonuclease associated with the double-stranded RNA binding protein DGCR8 to form the precursor miRNA (pre-miRNA) by cleaving the nucleotides on both sides of the hair pin. Pre-miRNAs are exported by exportin 5 into the cytoplasm. The stem-loop miRNA is then processed by the Dicer RNase III endonuclease to produce the double-strand mature miRNA. The mature miRNA is associated with Ago2 to form the RNA-induced silencing complex (RISC), which prevents target mRNAs expression in a specific manner relative to the stability of the bound of the RISC complex at 3′UTR on the target mRNA [5]. When the sequences are perfectly matched, the RISC complex is bound tightly to the mRNA; and the mRNA is degraded by the enzyme Ago2. When the sequences are not perfectly matched, the RISC complex inhibits the translation of the mRNA without degradation. The two different pathways lead to the same final outcome: a decrease in the protein level of the target gene [7]. RISC complex can be bound efficiently not only on the 3′UTR but also on the 5′UTR of the target mRNA to inhibit the translation [6]. The binding with 3′UTR has been well studied; however the binding with 5′UTR needs further investigation to better understand this regulation. Interestingly, recent studies have shown that animal miRNAs are able not only to repress, but also to activate gene expression by acting on mRNA stability and translational regulation [8]. The binding of RISC complex on 5′UTR is a necessary condition for the translation activation. It has been shown that the association of miRNAs with 5′UTR generally induces translation activation rather than repression [9].

According to the miRNA database, today 2578 mature and 1872 precursor forms of miRNAs have been identified in humans [10]. One of the first identified characteristics of the miRNAs is the very well conserved sequences in all species and these are expressed in tissues, in a specific manner [11]. miRNAs are critical in cell development, proliferation, communication, and tissue differentiation. They have been involved in regulating pregnancies [5]. Aberrant miRNA expression patterns have been found in pathologies and physiological processes including pregnancy and angiogenesis [12].

## 3. MicroRNA Functions in Human Placental Development

Development of the human placenta is critical for embryonic development and successful pregnancy. Differentiation, migration, invasion, angiogenesis, proliferation, and apoptosis mechanisms play an important role in key processes of placental development [13, 14]. The first step begins with the implantation of the blastocyst followed by the formation of various trophoblast cell types from the outer epithelial layer of the blastocyst, the trophectoderm [13]. Primitive syncytium is generated from the invasive trophoblast cell type migrating into the maternal endometrium. After the formation of the lacuna system, which is the ancestor of the intervillous space, cytotrophoblasts emanating from the trophectodermal layer produce primary villi by proliferation and invasion through the primitive syncytium. These primary villi change into secondary and tertiary villi characterized by the invasion of extraembryonic mesenchymal cells, villous branching, and vascularization. The cytotrophoblast fuses into multinucleated syncytiotrophoblasts which are eventually in close contact with placental vessels allowing efficient nutrient uptake by the fetus [14].

Studies have shown that miRNAs are produced in the human placenta and their expression is regulated by environmental factors such as hypoxia, signaling pathways, and epigenetic modification [15–17]. MicroRNAs are expressed in different ways during the various stages of placental development relative to their functions in regulating placental development and trophoblast cell activities [12]. miRNAs regulate placental development and functions through trophoblast cell proliferation, apoptosis, migration, invasion, and angiogenesis: miR-378a-5p [18], miR-376c [19], and miR-141 [20] enhance trophoblast cell proliferation whereas miRNA-155 [21] and miRNA-675 [22] inhibit this process. Apoptosis of trophoblast cells is induced by miR-29b [23] and inhibited by miR-182 [24]. It has been shown that miRNAs are involved in trophoblast cell migration and invasion: miR-376c [19] and miR-378a-5p [18] enhance trophoblast migration and invasion; miR-195 [25] and miR-21 [26, 27] promote trophoblast invasion. Conversely, miR-210 [28], miR-34a [29], and miR-29b [23] reduce trophoblast cell invasion; miR-155 [30] has an inhibiting effect on trophoblast cell migration. In placental angiogenesis, the abundant expression of Dicer in the perivascular villous stroma suggests that miRNAs play a role in placental vascularization and spiral artery remodeling [31]. It has been shown that miR-16 and miR-29b reduce placental angiogenesis [12].

More than 500 miRNAs are expressed in the human placenta which exhibits a specific miRNA expression pattern [11]. Numerous miRNAs, which are predominantly or exclusively expressed during pregnancy, are clustered in chromosomal regions and work synergistically [32]. The three most significant miRNA clusters are C19MC and miR-371-3 cluster (located on chromosome 19) and C14MC (located on chromosome 14) [33]. C19MC and C14MC are specifically expressed in the placenta [26, 34]. C19MC is the largest miRNA cluster identified with 46 pre-miRNAs transcribed only in the placenta [35] and expressed from the paternal allele [36]. C14MC contains 46 miRNAs and is encoded by maternally imprinted genes [17]. miR-371-3 cluster is adjacent to C19MC and contains 3 miRNAs. The expression of C19MC and miR-371-3 miRNAs increases from the first to the third trimester while C14MC miRNAs decrease during the same period [33]. These different expressions suggest that the placental miRNAs have specific functions at the various stages of the pregnancy.

Despite these interesting findings, the majority of placental miRNAs remain unknown. Only a small number of placental specific miRNAs have been studied for their role in placental development. Hence further research is required in this study.

## 4. Circulating Placental MicroRNA

In 1997, the discovery by Lo et al. of fetal cell free DNA in the plasma of pregnant women has led to the development of noninvasive diagnostic methods based on maternal blood for clinical applications such as fetal rhesus D genotyping, fetal sex determination, and recently fetal chromosomal aneuploidy detection [37]. With the discovery of miRNAs expression in placental tissue, new molecules with a strong diagnostic potential role as biomarkers for pregnancy-related diseases should be considered, inasmuch as circulating miRNAs have been detected in maternal plasma [3]. The absolute concentration of fetal DNA increases with the pregnancy as the fetus and the placenta grow [38]. Yet, it has been outlined that circulating miRNA concentration varies depending on the type of miRNA during the three trimesters of pregnancy [39, 40]. Therefore, the noninvasive circulating placental miRNA quantification for prenatal diagnosis should be performed in a specific time of the gestational age, preferably during the first trimester for early treatment or management of complicated pregnancies.

**Figure 2:**
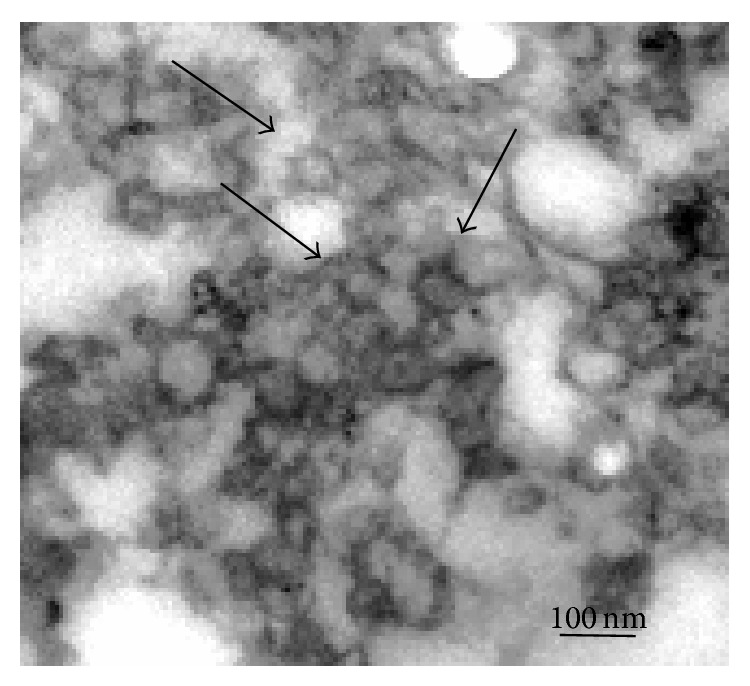
Isolated exosomes analyzed on electron microscopy.

MicroRNAs are exported from placental syncytiotrophoblasts and released into the maternal blood flow via exosomal nanoparticles [41]. Exosomes are small 60–80 nm membrane vesicles that are secreted by a multitude of cell types as a consequence of fusion of multivesicular lysosomes/late endosomes with the plasma membrane. Depending on their original tissue, exosomes can come into play in different physiological processes [42]. Exosomes originating from the placenta have played a crucial role in setting up an immune privilege for the developing fetus. Compared to nonpregnant women, exosomes are found in larger quantities in pregnant women [43]. Exosomes can be isolated from blood sample, for* in vivo* or* in vitro* studies from specific tissues of the human cell line performed by ultracentrifugation, and then visualized by electron microscopy [44] (Figure 2). In addition to the exosomal pathway, extracellular miRNAs derived from human trophoblasts can be released from the trophoblast layers in other forms, like microvesicles, apoptotic bodies, and protein-bound miRNAs [45]. Circulating miRNAs have been found to be complexed with circulating ribonucleoprotein and high-density lipoproteins [45, 46]. The extracellular form of miRNAs gives them relative stability and protection from digestion by RNase in human blood [41] thus reinforcing these small molecules as potential biomarkers.

## 5. Identification of Specific Circulating MicroRNA Expression Linked to Placental Pathology

Placental miRNAs are instrumental in placentation mechanisms such as trophoblast proliferation, syncytialization, and invasion. This has impact upon the major human placental diseases such as preeclampsia [47], intrauterine growth restriction [48], and early pregnancy loss [49]. The misregulated expression of placental circulating miRNAs has been detected in maternal blood with pregnancy complications (Table 1).

To identify miRNA biomarkers for noninvasive diagnosis on maternal blood of specific placental diseases, the first step is to describe precisely the expression of miRNAs in both normal and pathological human placentae of a specific disease using microarray or next generation sequencing methods to characterize the misregulated miRNAs in the pathology: the miRNAs are either under- or overregulated. Once the miRNAs have been determined as either under- or overregulated, they are studied by real-time PCR to establish the repeatability of the results both in the placenta and the maternal blood to identify biomarkers for clinical application [4]. A few expression studies have shown that the expression of miRNAs may not be the same in the placenta and in the maternal blood for certain pathologies. However other studies have shown that miRNAs in plasma or in the placenta increase or decrease at the same rate [47, 48].

The human placenta is a highly invasive and proliferative structure. Successful placentation is the one and only way to establish optimal blood and nutrient supply for normal growth. The invasive capacity is similar to cancer cell invasion with which it shares similar mechanisms. Therefore, miRNAs involved in human cancer and referred to as oncomiRs can be analyzed to suggest important miRNAs for placentation [49].

## 6. MicroRNA as Noninvasive Biomarkers in Preeclampsia

Preeclampsia (PE) is a major cause of maternal and fetal morbidity or mortality, preterm birth, and intrauterine growth restriction. This disease is characterized by maternal high blood pressure. If the systolic blood pressure is greater than 140 mmHg, the diastolic blood pressure is greater than 90 mmHg, and proteinuria occurs after 20 weeks' gestation, then one can diagnose the mother with preeclampsia. [47]. Risk factors for preeclampsia are maternal age, primiparity, previous preeclampsia, multiple fetuses during the same pregnancy, diabetes, high blood pressure, kidney disease, autoimmune diseases, the antiphospholipid syndrome, obesity, previous history, and ethnicity [47]. Preeclampsia is often diagnosed in the third trimester of the pregnancy; but the disorder is present long before the clinical symptoms appear [50]. Despite the lack of therapeutic treatment, predicting preeclampsia is a state-of-the-art method for managing pregnancy and improving the health of both the mother and the fetus. At present, there are no reliable biomarkers for early detection of preeclampsia because most of them are not sensitive or specific enough for clinical diagnosis [51]. To enhance the predictive value of the diagnosis, it is necessary that new biomarkers be found. Several studies have placed emphasis on proteins and metabolites in biofluids [51] but few have been concerned with miRNAs, which are yet becoming a promising class of biomarkers. Some studies have compared miRNAs expression on placenta between normal pregnancies and pregnancies with minor and severe preeclampsia; these studies have led to the identification of specific miRNAs involved in preeclampsia. Misregulated miRNAs were analyzed in maternal plasma for researching noninvasive biomarkers. MiR144 is significantly underexpressed both in severe and minor preeclampsia when compared with normal control [47]. Misregulation cases of miRNAs circulating in the plasma are more frequent in minor than in severe preeclampsia as opposed to the placenta tissue. These dysregulated miRNAs circulating in the plasma may be associated with the early pathological preeclampsia related changes. Another miRNA, miR-210, has been found to significantly increase in the plasma of preeclampsia-affected women [50].

Preeclampsia is a consequence of inadequate placental cytotrophoblast invasion, trophoblast invasion, and maternal spiral artery remodeling. These lead to reduced uteroplacental perfusion pressure and oxygen availability which in turn results in placental ischemia and hypoxia [52]. miR-144 is an important regulator in ischemia and hypoxia [47] and miR-210 is induced by hypoxia and regulated by transcriptional factor HIF-1 (hypoxia inducible factor) and NF-*κ*B [53]. Moreover, another research has shown that miR-210 inhibits trophoblast invasion [50]. These findings and the above-mentioned ones show that miR-144 and miR-210 play an important role in the pathogenesis of PE and that they might be useful biomarkers for the diagnosis of preeclampsia.

## 7. MicroRNAs as Noninvasive Biomarkers in Intrauterine Growth Restriction

Intrauterine growth restriction (IUGR), also known as fetal growth restriction (FGR), is characterized as birth weight less than the 10th percentile of the expected weight relative to the gestational age and the sex of the fetus. IUGR increases the risk of perinatal complications considerably [54]. IUGR has been traced to a malfunction of the placenta along with uteroplacental insufficiency at the interface between fetal and maternal circulation [55]. The uteroplacental insufficiency leads to deteriorated placenta transport of nutrients, mainly amino acids, lipids [56], and micronutrients such as iron and folate [57]. Preeclampsia, aneuploidies, or any genetic syndrome is major risk factors of IUGR. The mother's dietary deficiencies, her exposure to environmental factors, and placental epigenetic modifications are additional risk factors [58].

The role of miRNAs in IUGR has not yet been clearly understood compared to preeclampsia. However, we know that miR-141 contributes to IUGR by regulating pleomorphic adenoma gene 1 (*PLAG1*) expression [59]. The expression level of miR-141 in the placenta of pregnancies with IUGR is 3.4 times greater than that in normal placentae. Besides, the expression level of the miR-141 target gene, that is,* PLAG1*, decreases significantly in the IUGR placental tissue compared to controls. PLAG1 is mainly expressed in the placenta during pregnancy [59]. Another miRNA, miR-424, is a critical mediator of oxygen-dependent miRNAs; it is physiologically overregulated in placentae undergoing abnormal vascular development. Increased levels of miR-424 in placenta with IUGR have been found [60].

Local placental miRNAs such as mir-518b, mir-1323, mir-516b, mir-515-5p, mir-520h, mir-519d, and mir-526b are significantly lower in placentae in IUGR pregnancies compared to normal ones. However the corresponding circulating miRNA level does not change significantly [55]. This discrepancy has also been found for specific miRNAs regulated by hypoxia (miR-27a, miR-30d, miR-141, and miR-200c). The same observations are applicable to the following ubiquitous miRNA species (miR-205, miR-424, miR-451, and miR-491) and miRNAs from cluster C19MC known to be expressed mainly by the placenta (miR-517a, miR-518b, miR-518e, and miR-524) [48]. However, the overall level of these twelve specific miRNAs increases in the plasma of women with IUGR complicated pregnancies. The difference in misregulation level of the miRNAs between the plasma and the placenta may be indicative of placental injury in IUGR; placental injury lowers miRNA biogenesis while increasing the release of miRNAs onto the plasma [48].

Another high level of pregnancy-related extracellular miRNAs (miR-516-5p, miR-517, miR-518b, miR-520a, miR-520h, miR-525, and miR-526a) has been observed at the early stage of the pregnancy (between the 12th and the 16th weeks of gestation) in late-onset IUGR as opposed to normal pregnancies [39]. What has already been discovered needs to be completed to find reliable biomarkers for noninvasive diagnosis of IUGR on maternal plasma.

## 8. MicroRNAs as Noninvasive Biomarkers in Early Pregnancy Loss

The fetal loss before 20 weeks of pregnancy is called early pregnancy loss or miscarriage. This is a common event which complicates up to 15% of pregnancies [61]. Mechanisms leading to early pregnancy loss from noncytogenetic causes are related to defective trophoblast growth and impaired invasiveness by trophoblast cells leading to defective placentation [62].

Few studies have focused on miRNA implication and misregulation in early pregnancy loss. miR-17 and miR-19a are strikingly underregulated in the placenta when it comes to early spontaneous miscarriage [49]. This suggests that the above-mentioned miRNAs may play a critical role in early placentation. These results obtained from placenta samples have yet to be confirmed for maternal plasma to identify noninvasive miRNA biomarkers in early pregnancy loss.

## 9. Conclusion

miRNAs are relatively stable and expressed specifically depending on the various tissues. This makes them ideal candidates for diagnosis. The close link between altered expression of miRNAs in maternal plasma and pathological pregnancies has been well described.

Circulating miRNAs provide a new fetal genetic material easily collectable from maternal blood samples. They offer new potential for the development of noninvasive prenatal diagnostic tests.

Implementing noninvasive prenatal diagnosis using miRNAs involves the analysis of the misregulated miRNAs linked to the pathology. Under- or overexpressed miRNAs can be analyzed by relative quantification using real-time PCR. Important points need to be included when reporting investigation of cell-free microRNAs such as sample collection, miRNA isolation and quantification, and the normalization methods [63]. As for the screening of Down syndrome in maternal serum [64], the design of noninvasive tests to anticipate pregnancy complications should include results with multiple of the median (MoM) for each miRNA analyzed for measuring the level of the misregulation. Additionally, a risk should be calculated from biological and clinical parameters. National or international studies should be performed to determine the cut-off risk to conclude to a low or high risk for developing the pathology.

Discoveries of potential miRNAs for noninvasive biomarkers should be improved by deeper analyses and appropriate normalization methods to confirm the results obtained prior to application for clinical care. The levels of circulating miRNAs change relative to the gestational age. Studies have to include results for each trimester of the pregnancy. Then, microRNAs circulating in the maternal plasma might have the potential to be noninvasive diagnostic and prognostic biomarkers for pregnancy monitoring. They might prevent the development of gestational disorders and form the basis of personalized therapeutic strategies.

## Figures and Tables

**Table 1:** Altered expression of circulating placental miRNA in maternal blood with pregnancy complication for potential application in noninvasive diagnosis.

Pregnancy complication	miRNA upregulated	miRNA downregulated	References
Preeclampsia	miR-210		[50]
		miR-376c	[19]
	miR-24, miR-26a, miR-103, miR-130b, miR-181a, miR-342-3p, miR-574-5p		[65]
		miR-144	[47]

IUGR	miR-516-5p, miR-517, miR-518b, miR-520a, miR-520h, miR-525, miR-526a		[39]
	miR-27a-1, miR-30d, miR-93, miR-141, miR-200c, miR-205, miR-224, miR-335, mir-424, miR-451, miR-491		[48]

Early pregnancy loss		miR-17, miR-19a Found only on placental tissue To be validated on maternal blood	[49]
